# Breast cancer worry in higher-risk women offered preventive therapy: a UK multicentre prospective study

**DOI:** 10.1007/s10549-021-06183-x

**Published:** 2021-03-17

**Authors:** Kelly E. Lloyd, Louise H. Hall, Lucy Ziegler, Samuel G. Smith, ENGAGE investigators, Vanessa Adamson, Sarah Ainsworth, Malin Akerlund, Ivanna Baker, Julian Barwell, Jayne Beesley, Lisa Brock, Chrissie Butcher, Janice Carpenter, Martyn Clark, Shirley Cocks, Veronica Conteh, Martina Coulding, Sue Darby, Angela Duckworth, Gareth Evans, Catherine Fensom, Julie Fletcher, Kate Foster, Sara Grieg, Elaine Gullaksen, Jana Gurasashvili, Lisa Hardstaff, Rachel Hart, Kathryn Hoare, Jonathan Hoffman, Christopher Holcombe, Lynne Horton, Antony Howell, Farah Islam, Emma Jenkinson, Karen Jewers, Manisha Joshi, Amy Kirkby, Peter Kneeshaw, Natalie Knife, Jalal Kokan, Jin Li, Nicola Lunt, Douglas Macmillan, Karen Makinson, Evangelos Mallidis, Sarah Manyangadze, Charity Masvaure, Raksha Mistry, Alice Ngumo, Jane Ooi, Ashraf Patel, Vanessa Pope, Laura Price, Fiona Rabson, Lisa Richardson, Stephanie Ridgway, Karen Riley, Lorraine Roberts, Janet Ryan-Smith, Vian Salih, Nicky Scott, Mike Shere, Andrew Sloan, Nita Solanky, Amanda Taylor, Dinesh Thekkinkattil, Heather Thomas, Mangesh Thorat, Barbara Townley, Jayant S. Vaidya, Lynda Wagstaff, Shane Walsh, Lynsey Waring, Donna Watterson, Charlotte Westley, Lesley Wilkinson, Nicola Willis, Julia Wiseman

**Affiliations:** Leeds Institute of Health Sciences, University of Leeds, Worsley Building, Clarendon Way, Leeds, LS2 9NL UK

**Keywords:** Preventive therapy, Chemoprevention, Decision-making, Cancer worry

## Abstract

**Purpose:**

Women’s worry about developing breast cancer may influence their decision to use preventive therapy. However, the direction of this relationship has been questioned. We prospectively investigated the relationship between breast cancer worry and uptake of preventive therapy. The socio-demographic and clinical factors associated with high breast cancer worry were also investigated.

**Methods:**

Women at increased risk of developing breast cancer were recruited from clinics across England (*n* = 408). Participants completed a survey on their breast cancer worry, socio-demographic and clinical factors. Uptake of tamoxifen was recorded at 3 months (*n* = 258 women, 63.2%). Both primary and sensitivity analyses were conducted using different classifications of low, medium and high worry.

**Results:**

39.5% of respondents reported medium breast cancer worry at baseline and 21.2% reported high worry. Ethnic minority women were more likely to report high worry than white women (OR = 3.02, 95%CI 1.02, 8.91, *p* = 0.046). Women educated below degree level were more likely to report high worry than those with higher education (OR = 2.29, 95%CI 1.28, 4.09, *p* = 0.005). No statistically significant association was observed between worry and uptake. In the primary analysis, fewer respondents with medium worry at baseline initiated tamoxifen (low worry = 15.5%, medium = 13.5%, high = 15.7%). In the sensitivity analysis, participants with medium worry reported the highest uptake of tamoxifen (19.7%).

**Conclusions:**

No association was observed between worry and uptake, although the relationship was affected by the categorisation of worry. Standardised reporting of the classification of worry is warranted to allow transparent comparisons across cohorts.

**Supplementary Information:**

The online version contains supplementary material available at 10.1007/s10549-021-06183-x.

## Introduction

Breast cancer incidence is rising worldwide [1]. There is increasing interest in preventive therapy for people at higher risk of cancer as part of prevention efforts [2, 3]. The UK National Institute of Health and Care Excellence (NICE) considers women with a lifetime breast cancer risk of 17–30% to be at moderately high risk, and those exceeding 30% to be high risk [4, 5]. In a meta-analysis of nine randomised control trials, selective oestrogen receptor modulators (SERMs) reduced the incidence of breast cancer by at least 30% among women at increased risk [6]. In 2013, NICE published clinical guidelines (CG164) recommending SERMs (e.g. tamoxifen) are offered to higher-risk women with a family history of breast cancer [4].

Following the introduction of the NICE guidance (CG164), a UK prospective study reported low uptake of tamoxifen (14.7%) among women at increased risk of breast cancer [7]. Worry about developing breast cancer may influence women’s decision to use preventive therapy. Previous cross-sectional studies found women reporting higher levels of breast cancer worry were more likely to be interested in using preventive therapy [8, 9]. Prospective US studies have also observed an association between breast cancer worry and uptake of preventive therapy [10, 11]. These findings suggest women who worry more about developing breast cancer may be more likely to use preventive therapy. However, there are mixed results on the direction of this relationship [12].

There may be an inverted U-shaped relationship between breast cancer worry and uptake of preventive therapy, whereby moderate levels of worry are most strongly associated with uptake. An inverted U-shaped relationship has been observed between breast cancer worry and other cancer protective behaviours, such as screening [13–16]. These findings suggest people with moderate worries about developing breast cancer are more likely to engage with preventive behaviours, compared with those reporting lower or higher worries. Few prospective studies have investigated whether there is a U-shaped relationship between breast cancer worry and uptake of preventive therapy. Given the negative psychological side effects of worry [17], identifying those who experience cancer-related worry is warranted. Previous data also indicate people from lower socioeconomic status (SES) backgrounds [18], and ethnic minority respondents [18, 19] are more likely to experience higher levels of cancer worry.

We assessed levels of breast cancer worry among women considering preventive therapy in a UK clinical practice setting and investigated whether there was an association between worry and uptake. We also investigated the socio-demographic and clinical factors associated with higher worry, and assessed whether worry changed over time. This analysis is part of the ENGAGE study; a multicentre prospective investigation of preventive therapy uptake in England among women at increased risk of breast cancer [7, 20].

## Methods

### Recruitment

Twenty clinics in England supported recruitment between September 2015 and December 2016. This included family history clinics (*n* = 12), breast clinics (*n* = 4), clinical genetic centres (*n* = 3) and a family history clinic with genetics support (*n* = 1). After a clinic appointment, a healthcare professional approached the woman to discuss the study. Women who verbally consented were given a study pack containing the baseline survey. Informed consent was implied following the return of the survey. An identification code was assigned to participants who verbally consented, which was linked to their baseline survey. After verbal consent, women’s personal data were sent to the study team via a secure online portal. These data included: patients age, email address, home address and their risk classification (‘moderately high’ or ‘high’). At 3 months, baseline survey respondents were sent a follow-up survey.

The ENGAGE study was granted ethical approval by the National Research Ethics Service Committee North West—Preston (14/NW/1408). Women were eligible to participate if they were over the age of 18, classified as ‘moderately high’ or ‘high’ risk of breast cancer, spoke English, had discussed preventive therapy with a healthcare professional and had no known contraindications for tamoxifen use. Women were excluded if they were unable to consent or had a previous breast cancer diagnosis.

### Measures

The baseline survey contained measures of socio-demographic and clinical factors, and breast cancer worry. The follow-up survey measured uptake of tamoxifen and breast cancer worry at 3 months. The time period between the baseline and follow-up survey was decided on the basis that this was a reasonable amount of time for participants to consider the harms and benefits of tamoxifen, and to have spoken with a GP about obtaining a prescription.

#### Socio-demographic and clinical factors

The baseline survey contained items on demographic and socioeconomic factors, some of which were dichotomised in the analysis. These measures included nulliparity (yes vs. no), marital status (married/cohabiting vs. single/divorced/separated/widowed), education level (≥ degree level vs. < degree level), ethnicity (white ethnic groups vs. ethnic minority groups), employment status (full-time vs. all other employment types) and self-reported health (poor, fair, good, excellent). Age was calculated from date of birth provided from National Health Service (NHS) records, with age recoded to ≤ 35 years; 36–49 years and; ≥ 50 years. Postcodes were used to calculate a participant’s Index of Multiple Deprivation (IMD) score, and women were classified into tertiles of neighbourhood deprivation (low, middle and high) [21]. Breast cancer risk category, based on the NICE guideline CG164 [4], was provided by staff at the clinic they had attended.

#### Breast cancer worry

Two items measured breast cancer worry, which were adapted from the Lerman Breast Cancer Worry Scale [16]. Respondents were asked to assess how frequently each statement was true for them over the past 7 days. The statements were ‘I worried about developing breast cancer’ and ‘My worries about breast cancer interfered with my daily activities’. Each item was assessed on a 4-point Likert scale: ‘Not at all’, ‘Rarely’, ‘Sometimes’ and ‘Often’. Women were classified as reporting low/medium/high worry using a method adapted from Zhang et al. [14]. Participants reporting ‘Not at all’ or ‘Rarely’ to both worry questions were classified as low worry. Participants reporting ‘Sometimes’ to either or both questions were classified as medium worry, and participants reporting ‘Often’ to either or both questions were classified as high worry. The breast cancer worry scale was administered to participants at baseline and 3-month follow-up. The Cronbach’s alpha for the scale was 0.73.

#### Preventive therapy decision

At 3 months, we measured self-reported uptake of tamoxifen. Women were asked to tick which of the following categories applied to them: (1) ‘I decided immediately that I did not want to take tamoxifen’; (2) ‘After some thought, I decided that I did not want to take tamoxifen’; (3) ‘I am still deciding if I want to take tamoxifen’; (4) ‘I met with my GP to talk about tamoxifen, and I decided against it’; (5) ‘I met with my GP to talk about tamoxifen, but they would not prescribe it’; (6) ‘I have a prescription for tamoxifen from my GP’; (7) ‘I am currently taking tamoxifen’. Women were considered to be using tamoxifen if they ticked categories 6 or 7, with responses 1–5 indicating no uptake.

### Data analysis

The socio-demographic and clinical factors were compared between responders and non-responders to the baseline questionnaire, and responders and non-responders to the 3-month questionnaire using Pearson’s Chi-square test. Responses on the baseline worry scale were compared between responders and non-responders to the 3-month questionnaire using a between-subjects *t* test.

We used Pearson’s Chi-square test to assess for differences in baseline breast cancer worry (‘low/medium’ vs. ‘high’) across each of the socio-demographic and clinical factors. Fisher’s exact test was used if > 20% of cells had an expected count < 5. We conducted a multivariable logistic regression model, including all socio-demographic and clinical variables to identify factors associated with high breast cancer worry at baseline (vs. ‘low/medium’ worry).

Univariable and multivariable logistic regression models examined whether women’s baseline breast cancer worry (‘low’, ‘medium’ or ‘high’) was associated with uptake of tamoxifen at 3 months. The multivariable model was adjusted for all socio-demographic and clinical factors. We used a McNemar test to assess for differences in baseline and 3-month worry across the three different worry groups. We used a within-subjects t test to compare mean worry scores between baseline and 3-month follow-up.

Sensitivity analyses were conducted to assess if the approach used to classify the worry groups affected the study’s findings [22]. In the sensitivity analysis, participants reporting ‘Not at all’ to both worry questions were classified as low worry. Participants reporting ‘Rarely’ to either or both worry questions were classified as medium worry, and participants reporting ‘Sometimes’ or ‘Often’ to either or both worry questions were classified as high worry.

The analysis plan for the study was pre-registered (https://osf.io/6dupm). The analysis was conducted in SPSS version 25, with statistical significance set at a 2-sided *p* < 0.05.

## Results

In total, 732 women consented to participate. For the baseline survey, 408 (55.7%) women responded, and 258 (63.2%) women returned the 3-month follow-up survey. Among the 408 baseline respondents, 275 (67.4%) attended a family history clinic, 49 (12.0%) attended a breast clinic, 25 (6.1%) attended a clinical genetic centre, and 59 (14.5%) attended a family history clinic with genetics support. Participant characteristics are presented in Table 1.

**Table 1:** Description of participants demographic, clinical and breast cancer worry characteristics at baseline (*n* = 408)

Demographic, clinical and worry factors	Baseline respondents
Age, mean ± SD	45.30 ± 7.82
Age, *n* (%)	
≤ 35 years	41 (10)
36–49 years	259 (63.5)
≥ 50 years	108 (26.5)
Children, *n* (%)	
Yes	314 (77.0)
No	94 (23.0)
Ethnic group, *n* (%)	
White ethnic groups	384 (95.5)
Ethnic minority groups	18 (4.5)
Caribbean	3
Indian	5
Pakistani	2
Other Asian	1
White and Black Caribbean	2
White and Black African	1
Other mixed ethnicity	2
Any other	2
Missing, *n*	6
Education level, *n* (%)	
Degree or above	176 (44.2)
Below degree level	222 (55.8)
Missing, *n*	10
Health status, *n* (%)	
Poor	16 (4.0)
Fair	78 (19.5)
Good	240 (60.0)
Excellent	66 (16.5)
Missing, *n*	8
Risk level, *n* (%)	
Moderate	243 (59.6)
High	159 (39.0)
Unclear	6 (1.5)
SES, *n* (%)	
Low (most deprived)	120 (29.9)
Middle	131 (32.7)
High (least deprived)	150 (37.4)
Missing, *n*	7
Employment, *n* (%)	
Full-time	348 (85.3)
All other employments	60 (14.7)
Marital status, *n* (%)	
Married or cohabiting	298 (74.3)
Unmarried	103 (25.7)
Missing, *n*	7
Breast cancer worry, *n* (%)	
Low worry	159 (39.3)
Medium worry	160 (39.5)
High worry	86 (21.2)
Missing, *n*	3
Worry about developing breast cancer, *n* (%)	
Not at all	52 (12.8)
Rarely	108 (26.7)
Sometimes	159 (39.3)
Often	86 (21.2)
Missing, *n*	3
Worries interfered with daily activities, *n* (%)	
Not at all	267 (65.9)
Rarely	84 (20.7)
Sometimes	40 (9.9)
Often	14 (3.5)
Missing, *n*	3

*SES* socioeconomic status, *SD* standard deviation

There were no significant differences between responders to the baseline survey (408 women) and non-responders (324 women) with regard to clinical risk (*p* = 0.62), socioeconomic status (*p* = 0.054) or age (*p* = 0.086). Baseline respondents retained at 3 months were more likely to be from a higher socioeconomic status (SES) group (*p* < 0.001). There were no significant differences in the mean baseline worry scores between responders who only responded to the baseline survey compared with those who completed both the baseline and 3-month survey (*p* = 0.234).

### Breast cancer worry and participant characteristics

In total, 408 women provided baseline worry data. Among these, 39.3% had low baseline breast cancer worry, 39.5% had moderate worry, and 21.2% had high worry. For each of the worry items, 21.2% worried about developing breast cancer ‘often’, while only 3.5% felt their worries about breast cancer ‘often’ interfered with their daily activities (Table 1).

In a multivariable logistic regression model (Table 2), women from an ethnic minority group were more likely to report high breast cancer worry than white women (OR = 3.02, 95% CI 1.02, 8.91, *p* = 0.046). Women who were educated to below degree level were more likely to report high worry than women educated to degree level or above (OR = 2.29, 95% CI 1.28, 4.09, *p* = 0.005).

**Table 2:** Breast cancer worry by participant characteristics and Pearson’s Chi-square test and multivariable logistic regression model (*n* = 385)

High breast cancer worry (*n*; %)		Chi-square		Multivariable	
		*Χ*^2^	*P* value	OR (95% CI)	*P* value
Age		1.21	0.546		
≤ 35 years	6 (14.6)			0.58 (0.17–2.02)	0.393
36–49 years	57 (22.2)			1.40 (0.77–2.60)	0.273
≥ 50 years	23 (21.5)			Ref	
Children		0.20	0.656		
Yes	68 (21.7)			1.11 (0.56–2.21)	0.773
No	18 (19.6)			Ref	
Ethnic group		6.28	0.032*		
White	76 (19.8)			Ref	
Ethnic minority	8 (44.4)			3.02 (1.02–8.91)	**0.046**
Education level		9.61	**0.002**		
Degree or above	25 (14.2)			Ref	
Below degree level	60 (27.0)			2.29 (1.28–4.09)	**0.005**
Health status		3.18	0.204		
Poor/fair	25 (26.6)			1.43 (0.58–3.51)	0.434
Good	49 (20.4)			1.13 (0.50–2.53)	0.767
Excellent	10 (15.2)			Ref	
Risk level		2.93	0.219*		
Moderate	45 (18.7)			Ref	
High	39 (24.7)			1.24 (0.72–2.11)	0.440
Unclear	2 (33.3)			1.21 (0.19–7.87)	0.843
SES		7.30	**0.026**		
Low (most deprived)	32 (26.9)			1.89 (0.96–3.71)	0.066
Middle	30 (23.1)			1.79 (0.92–3.47)	0.085
High (least deprived)	21 (14.0)			Ref	
Employment		1.85	0.173		
Full-time	70 (20.1)			Ref	
All other employments	16 (28.1)			1.34 (0.65–2.80)	0.431
Marital status		0.43	0.514		
Married or cohabiting	61 (20.5)			0.69 (0.37–1.28)	0.236
Unmarried	24 (23.5)			Ref	

Bold text indicates statistical significance (*P* ≤ 0.05)

*Fisher’s exact test

We carried out a sensitivity analysis whereby participants who responded ‘Sometimes’ or ‘Often’ to either or both worry questions were categorised into the high worry group. In the sensitivity analysis, 52 (12.8%) women were categorised as having low worry at baseline. Most women (246; 60.7%) were categorised as having high worry, and 107 (26.4%) were categorised as moderate worry (Supplementary Table 1S). Ethnicity was no longer significantly associated with high worry in the sensitivity analysis (Table 2S); however, the effect size with education was similar (OR = 2.00, 95% CI 1.27, 3.16, *p* = 0.003). In addition, women with poor/fair (OR = 2.90, 95% CI 1.43, 5.92, *p* = 0.003) and good health status (OR = 1.80, 95% CI 1.00, 3.24, *p* = 0.050) were more likely to report high breast cancer worry than those with excellent health status.

### Uptake of preventive therapy and breast cancer worry

Among the 258 women who completed the 3-month follow-up questionnaire, 38 (14.7%) had initiated tamoxifen. A higher proportion of women in the low worry group (16/103; 15.5%) and high worry group (8/51; 15.7%) at baseline initiated preventive therapy, compared with the medium worry group (14/104; 13.5%). A multivariable logistic regression model examined the association of breast cancer worry and uptake, after adjusting for all socio-demographic and clinical variables. Breast cancer worry was not significantly associated with tamoxifen initiation in the multivariable model (Table 3). No socio-demographic factors were significantly associated with uptake in the multivariable model (Table 3).

**Table 3:** Uptake of tamoxifen by breast cancer worry and participant characteristics in the univariable and multivariable logistic regression model (*n* = 247)

Uptake of tamoxifen (*n*; %)		Univariable		Multivariable	
		OR (95% CI)	*P* value	OR (95% CI)	*P* value
Breast cancer worry					
Low worry	16 (15.5)	Ref		Ref	
Medium worry	14 (13.5)	0.85 (0.39–1.84)	0.672	0.87 (0.38–2.01)	0.744
High worry	8 (15.7)	1.01 (0.40–2.55)	0.980	1.07 (0.38–3.05)	0.899
Age					
≤ 35 years	1 (3.8)	0.28 (0.03–2.36)	0.242	0.36 (0.04–3.41)	0.373
36–49 years	29 (17.3)	1.46 (0.63–3.39)	0.378	1.30 (0.49–3.42)	0.597
≥ 50 years	8 (12.5)	Ref		Ref	
Children					
Yes	36 (17.6)	5.43 (1.26–23.34)	**0.023**	4.66 (0.99–21.99)	0.052
No	2 (3.8)	Ref		Ref	
Ethnic group					
White	37 (15.0)	1.41 (0.17–11.60)	0.750	1.91 (0.20–18.06)	0.572
Ethnic minority	1 (11.1)	Ref		Ref	
Education level					
Degree or above	20 (17.2)	1.41 (0.71–2.82)	0.327	1.83 (0.80–4.16)	0.152
Below degree level	18 (12.9)	Ref		Ref	
Health status					
Poor/fair	5 (10.6)	0.68 (0.20–2.32)	0.538	0.73 (0.20–2.67)	0.629
Good	25 (16.6)	1.13 (0.46–2.82)	0.787	1.24 (0.47–3.29)	0.666
Excellent	7 (14.9)	Ref		Ref	
Risk level					
Moderate	24 (15.1)	1.05 (0.52–2.15)	0.885	0.91 (0.42–1.95)	0.910
High	14 (14.4)	Ref		Ref	
Unclear	0	–	–	–	–
SES					
Low (most deprived)	7 (11.9)	0.78 (0.30–2.03)	0.613	1.13 (0.41–3.15)	0.814
Middle	14 (16.3)	1.13 (0.52–2.47)	0.759	1.71 (0.72–4.08)	0.227
High (least deprived)	16 (14.7)	Ref		Ref	
Employment					
Full-time	32 (14.5)	Ref		Ref	
All other employments	6 (16.2)	1.14 (0.44–2.96)	0.783	1.50 (0.54–4.18)	0.438
Marital status					
Married or cohabiting	33 (16.7)	2.16 (0.80–5.81)	0.127	1.67 (0.52–5.40)	0.389
Unmarried	5 (8.5)	Ref		Ref	

Bold text indicates statistical significance (*P* ≤ 0.05)

In the sensitivity analysis, we found that a higher proportion of women categorised as medium worry at baseline (13/66; 19.7%) initiated tamoxifen, compared with women categorised as high worry (22/155; 14.2%) and low worry (3/37; 8.1%). None of the worry levels were significantly associated with tamoxifen uptake (Table 3S).

### Breast cancer worry at baseline and 3 months

Breast cancer worry was compared between baseline and 3 months in the sample of women who provided both baseline and follow-up data (Table 4). A higher proportion of participants were categorised as high breast cancer worry at baseline (51/258; 19.8%), compared with the proportion categorised as high worry at the 3-month follow-up (32/248; 12.9%). A lower proportion of women were categorised as low worry at baseline (103/258; 39.9%) compared with 3 months (115/248; 46.4%).

**Table 4:** Proportion of women categorised as low, medium and high breast cancer worry at baseline and at 3-month follow-up, in the sample of women who provided both baseline and follow-up data (*n* = 258)

	Baseline (*n* = 258)	3 months (*n* = 248)
Breast cancer worry groups, *n* (%)		
Low worry	103 (39.9)	115 (46.4)
Medium worry	104 (40.3)	101 (40.7)
High worry	51 (19.8)	32 (12.9)

Missing worry data for ten respondents at 3-month follow-up

There was no significant difference in the proportion of women categorised as low, medium and high breast cancer worry at baseline compared with the worry categories at 3 months (*p* = 0.077). A within-subjects comparison of the average worry level indicated a significant decline in worry between baseline (*M* = 4.09, SD = 1.54) and 3 months (*M* = 3.72, SD = 1.57; *p* < 0.001).

In the sensitivity analysis, the proportion of women categorised as medium worry and high worry decreased between baseline (low worry = 14.3%, medium = 25.6%, high = 60.1%) and 3-month follow-up (low worry = 29.8%, medium = 16.5%, high = 53.6%; *p* < 0.001).

## Discussion

### Summary of findings

In this UK prospective study of women at increased risk of breast cancer, over one-fifth of women reported high worry immediately after an appointment to discuss their cancer risk. Breast cancer worry was not consistent over time. Higher worry was reported immediately after the appointment than at 3-month follow-up, although half of our sample maintained a medium or high level of worry at follow-up. Support for women considering the use of preventive therapy may be particularly needed among specific socio-demographic groups. Women from ethnic minority groups and women who had lower educational qualifications were more likely to report high breast cancer worry immediately following their appointment.

### Relation to wider literature and implications for practice

Women’s worries about developing breast cancer did not appear to affect their decisions regarding tamoxifen use. These results contrast with previous prospective studies, which observed that women who had high worry about developing breast cancer were more likely to initiate preventive therapy [10, 11]. We also investigated whether medium levels of worry were the optimal level to motivate women to initiate tamoxifen, as predicted by the C-SHIP model [23] and Yerkes–Dodson law [24]. Previous research found evidence supporting an inverted U-shaped relationship between worry and breast cancer screening attendance, whereby lower and higher worry was associated with reduced attendance [13–15]. We did not observe a U-shaped relationship between worry and uptake in the primary analysis.

There was some evidence to support the inverted U-shaped between worry and uptake in the sensitivity analysis, indicating that the interpretations of this relationship will be affected by how worry is categorised. When a higher proportion of respondents were categorised into the high worry group, the inverted U-shaped was more prominent. To assess the direction of this relationship in future research, standardised reporting of the classification of low, medium and high worry is warranted to allow transparent comparisons across cohorts. Additionally, there is a need for future studies to be pre-registered to increase the transparency of the intended analyses.

Women with lower educational qualifications, and those from ethnic minority groups were more likely to report high worry about developing breast cancer. These results are comparable to previous research that has identified similar inequalities in cancer worry in the UK general population [18, 19]. The findings indicate inequalities in cancer worry may extend to individuals at increased risk of developing the disease. It is unclear why these groups experience greater worry about breast cancer, but previous evidence suggests some ethnic minority groups may be more fearful of the social consequences of diagnosis [25]. Further research should examine the factors influencing cancer worry in higher-risk individuals from these socio-demographic groups. Future research should also investigate which ethnic minority groups are more likely to experience higher levels of cancer worry.

Women with lower educational qualifications may need additional support when offered preventive therapy. Women educated below degree level have previously been found to report lower levels of knowledge on the benefit and harms of tamoxifen [26]. The study also found women were more likely to initiate tamoxifen if they reported greater knowledge on its benefit and harms [26]. Decision aids may be more beneficial in improving health outcomes for disadvantaged patients, compared with those with higher literacy and from higher SES backgrounds [27]. A meta-analysis of randomised control trials found strong evidence to suggest cancer-related decision aids increase patients’ knowledge on the topic, without increasing anxiety [28]. Decision aids for preventive options have also been shown to reduce cancer-related distress in women at increased risk of breast cancer [29]. The development of a decision aid for preventive therapy could address knowledge and worry inequalities among women at increased risk of breast cancer.

Reducing patient’s breast cancer worries is important to increase their feelings of satisfaction with treatment decisions [10]. One approach is for clinicians to adopt an ‘affective communication’ style, for example, being emotionally supportive with regard to patients’ breast cancer-related worries when discussing their risk reducing options. An experimental study found that affective communication reduced patients’ feelings of anxiety and increased their ability to recall information on treatment options [30].

Although our study did not find evidence of an association between breast cancer worry and uptake of tamoxifen, there may be other influential factors affecting uptake. Our analysis was part of the ENGAGE study; a prospective investigation of the psychological, clinical and demographic factors associated with uptake of breast cancer preventive therapy [7, 20, 26]. Previous analyses of the ENGAGE cohort have observed women with children [7], and those reporting lower concerns about using tamoxifen [20] were more likely to initiate preventive therapy. Additionally, we previously found that women who felt more informed on tamoxifen were less likely to be still deciding on the medication at three month follow-up [26]. The findings from our ENGAGE study contribute to the wider literature on decision-making in the area of breast cancer preventive therapy. We also measured additional patient-reported outcomes that have not yet been reported, but have previously been found to be associated with increased uptake of preventive therapy. These include perceived risk of breast cancer [10] and intrusive (i.e. involuntary and repetitive) thoughts and feelings on breast cancer [10].

### Strengths and limitations

This was the first UK study to investigate breast cancer worry and uptake of tamoxifen in a routine clinical setting. This context is important as preventive therapy uptake is higher in trial settings [31]. Women were recruited from 20 different centres and four different clinic types across England, and recruitment was open for over one year. We used a prospective design, which overcomes many of the limitations of previous cross-sectional studies assessing cancer worry [8, 9, 32, 33].

This study had limitations. Uptake of preventive therapy was self-reported, which may have led us to overestimate this behaviour. While our sample size was reasonably large, the number of women initiating tamoxifen was low, which resulted in low precision for some comparisons. Similarly, only 18 ethnic minority participants were recruited, which reduces confidence in the associations between ethnicity, worry and uptake. Tamoxifen was the chemoprevention agent specified in the questionnaire, as the study was conducted before the introduction of anastrozole in 2017 [5]. Our findings should therefore be carefully generalised to women considering anastrozole. Additionally, we recruited women with a family history of breast cancer, and therefore our findings may not be generalisable to other potential users of tamoxifen, such as women with high-risk benign lesions.

Over 40% of women who verbally consented to participate in the study did not complete the baseline survey, which may have resulted in selection bias. While we did not find significant differences in clinical risk, socioeconomic status and age between responders and non-responders to the baseline survey, there may be other important differences in characteristics between these two groups that we did not measure (e.g. ethnicity and education level). There was also evidence of a selection bias among responders to the follow-up survey. Approximately two-thirds (63%) of participants completed the follow-up survey at three months, with respondents from higher SES more likely to return the follow-up questionnaire. This may have caused bias in the interpretation of our data, as there is evidence to suggest cancer preventive healthcare is underutilised by those from lower SES [34, 35]. Therefore, we may have overestimated rates of uptake of preventive therapy in this cohort. Reminder postcards and questionnaires were sent to participants to minimise dropout rates in our study; however, more intensive retention approaches may be needed to improve response rates among those at lower SES [36].

## Conclusion

In this UK prospective study of women at increased risk of breast cancer, most respondents reported medium to high levels of breast cancer worry following their initial appointment to discuss their cancer risk. However, worry was not linearly associated with uptake, nor was there a U-shaped association. Women with lower educational qualifications and ethnic minority women were more likely to report higher worry about developing breast cancer. Decision aids could be developed specifically for women from these socio-demographic groups, to reduce their feelings of cancer worry when offered preventive therapy. To advance the field, standardised reporting of the classification of low, medium and high worry is warranted, and pre-registered studies are required.

## Supplementary Information

Below is the link to the electronic supplementary material.

Supplementary file 1 (DOCX 19 kb)

## Data Availability

Participants did not provide explicit consent for their data to be shared in public repositories. Therefore, data may not be made publicly available due to ethical restrictions. We can share the anonymised version of the data to individual qualified researchers upon request. Data requests may be sent to the corresponding author of this paper. The surveys used in the study and the SPSS syntax of the analysis can be found at https://osf.io/mqz9y/.
