# Supplementary material for: Breast cancer worry in higher-risk women offered preventive therapy: a UK multicentre prospective study
Source: Breast Cancer Res Treat. 2021 Mar 17;188(3):703–12. doi: 10.1007/s10549-021-06183-x (PMC8720078; doi:10.1007/s10549-021-06183-x)
Supplement: Supplementary file 1 — (DOCX 19 kb) [file 10549_2021_6183_MOESM1_ESM.docx]

**Title:** Breast cancer worry in higher risk women offered preventive therapy: a UK multicentre prospective study

**Authors:** K.E. Lloyd^1^, L. H. Hall^1^, L. Ziegler^1^, S.G. Smith^1^, on behalf of the ENGAGE investigators.

**Affiliations:**

1. Leeds Institute of Health Sciences, University of Leeds, Leeds, UK

**Corresponding author:** Dr Samuel G. Smith, University of Leeds, Leeds Institute of Health Sciences, Worsley Building, Clarendon Way, Leeds, LS2 9NL, T: 0113 343 0892, E: [s.smith1@leeds.ac.uk](mailto:s.smith1@leeds.ac.uk)

**Journal:** Breast Cancer Research and Treatment

## Table 1S. Sensitivity analysis: Description of participants breast cancer worry at baseline (n = 408)

| **Demographic, clinical and worry factors** | **Baseline respondents** |
| --- | --- |

| **Breast cancer worry, n (%)** |  |
| --- | --- |
| Low worry | 52 (12.8) |
| Medium worry | 107 (26.4) |
| High worry | 246 (60.7) |
| Missing, n | 3 |

## Table 2S. Sensitivity analysis: Breast cancer worry by participant characteristics and chi-square test and multivariable logistic regression model (n = 385)

| **High breast cancer worry (n; %)** | | **Chi-square** | | **Multivariable** | |
| --- | --- | --- | --- | --- | --- |
|  |  | ***Χ*^2^** | **P-value** | **OR (95% CI)** | **P-value** |
| **Age** |  | 1.00 | 0.607 |  |  |
| ≤ 35 years | 22 (53.7) |  |  | 0.97 (0.41 – 2.08) | 0.943 |
| 36 – 49 years | 159 (61.9) |  |  | 1.28 (0.76 – 2.13) | 0.355 |
| ≥ 50 years | 65 (60.7) |  |  | Ref |  |
| **Children** |  | 0.49 | 0.484 |  |  |
| Yes | 193 (61.7) |  |  | 0.90 (0.50 – 1.60) | 0.708 |
| No | 53 (57.6) |  |  | Ref |  |
| **Ethnic group** |  | 1.02 | 0.312 |  |  |
| White | 231 (60.3) |  |  | Ref |  |
| Ethnic minority | 13 (72.2) |  |  | 1.93 (0.61 – 6.07) | 0.262 |
| **Education level** |  | 10.96 | **0.001** |  |  |
| Degree or above | 91 (51.7) |  |  | Ref |  |
| Below degree level | 151 (68.0) |  |  | 2.00 (1.27 – 3.16) | **0.003** |
| **Health status** |  | 11.34 | **0.003** |  |  |
| Poor/ Fair | 66 (70.2) |  |  | 2.90 (1.43 – 5.92) | **0.003** |
| Good | 147 (61.3) |  |  | 1.80 (1.00 – 3.24) | **0.050** |
| Excellent | 29 (43.9) |  |  | Ref |  |
| **Risk level** |  | 1.87 | 0.417* |  |  |
| Moderate | 141 (58.5) |  |  | Ref |  |
| High | 102 (64.6) |  |  | 1.25 (0.80 – 1.95) | 0.334 |
| Unclear | 3 (50.0) |  |  | 0.49 (0.09 – 2.58) | 0.397 |
| **SES** |  | 1.11 | 0.574 |  |  |
| Low (most deprived) | 69 (58.0) |  |  | 0.81 (0.47 – 1.40) | 0.447 |
| Middle | 83 (63.8) |  |  | 1.10 (0.65 – 1.85) | 0.734 |
| High (least deprived) | 88 (58.7) |  |  | Ref |  |
| **Employment** |  | 1.63 | 0.687 |  |  |
| Full-time | 210 (60.3) |  |  | Ref |  |
| All other employments | 36 (63.2) |  |  | 1.12 (0.58 – 2.13) | 0.743 |
| **Marital status** |  | 4.70 | **0.030** |  |  |
| Married or cohabiting | 191 (64.1) |  |  | 1.65 (0.97 – 2.78) | 0.063 |
| Unmarried | 53 (52.0) |  |  | Ref |  |

*Fisher’s Exact Test

## Table 3S. Sensitivity analysis: Uptake of tamoxifen by breast cancer worry and participant characteristics and univariable and multivariable logistic regression model (n = 247).

| **Uptake of tamoxifen (n; %)** | | **Univariable** | | **Multivariable** | |
| --- | --- | --- | --- | --- | --- |
|  |  | **OR (95% CI)** | **P-value** | **OR (95% CI)** | **P-value** |
| **Breast cancer worry** |  |  |  |  |  |
| Low worry | 3 (8.1) | Ref |  | Ref |  |
| Medium worry | 13 (19.7) | 2.78 (0.74 – 10.48) | 0.131 | 2.65 (0.66 – 10.58) | 0.168 |
| High worry | 22 (14.2) | 1.88 (0.53 – 6.63) | 0.330 | 1.86 (0.50 – 6.96) | 0.355 |
| **Age** |  |  |  |  |  |
| ≤ 35 years | 1 (3.8) | 0.28 (0.03 – 2.36) | 0.242 | 0.36 (0.04 – 3.50) | 0.380 |
| 36 – 49 years | 29 (17.3) | 1.46 (0.63 – 3.39) | 0.378 | 1.32 (0.50 – 3.49) | 0.580 |
| ≥ 50 years | 8 (12.5) | Ref |  | Ref |  |
| **Children** |  |  |  |  |  |
| Yes | 36 (17.6) | 5.43 (1.26 – 23.34) | **0.023** | 4.40 (0.93 – 20.77) | 0.061 |
| No | 2 (3.8) | Ref |  | Ref |  |
| **Ethnic group** |  |  |  |  |  |
| White | 37 (15) | 1.41 (0.17 – 11.60) | 0.750 | 2.02 (0.21 – 19.12) | 0.542 |
| Ethnic minority | 1 (11.1) | Ref |  | Ref |  |
| **Education level** |  |  |  |  |  |
| Degree or above | 20 (17.2) | 1.41 (0.71 – 2.82) | 0.327 | 1.70 (0.75 – 3.85) | 0.204 |
| Below degree level | 18 (12.9) | Ref |  | Ref |  |
| **Health status** |  |  |  |  |  |
| Poor/ Fair | 5 (10.6) | 0.68 (0.20 – 2.32) | 0.538 | 0.68 (0.18 – 2.50) | 0.556 |
| Good | 25 (16.6) | 1.13 (0.46 – 2.82) | 0.787 | 1.18 (0.44 – 3.16) | 0.744 |
| Excellent | 7 (14.9) | Ref |  | Ref |  |
| **Risk level** |  |  |  |  |  |
| Moderate | 24 (15.1) | 1.05 (0.52 – 2.15) | 0.885 | 0.90 (0.42 – 1.94) | 0.786 |
| High | 14 (14.4) | Ref |  | Ref |  |
| Unclear | 0 | - | - | - | - |
| **SES** |  |  |  |  |  |
| Low (most deprived) | 7 (11.9) | 0.78 (0.30 – 2.03) | 0.613 | 1.09 (0.39 – 3.05) | 0.870 |
| Middle | 14 (16.3) | 1.13 (0.52 – 2.47) | 0.759 | 1.78 (0.75 – 4.25) | 0.193 |
| High (least deprived) | 16 (14.7) | Ref |  | Ref |  |
| **Employment** |  |  |  |  |  |
| Full-time | 32 (14.5) | Ref |  | Ref |  |
| All other employments | 6 (16.2) | 1.14 (0.44 – 2.96) | 0.783 | 1.58 (0.56 – 4.43) | 0.386 |
| **Marital status** |  |  |  |  |  |
| Married or cohabiting | 33 (16.7) | 2.16 (0.80 – 5.81) | 0.127 | 1.78 (0.55 – 5.78) | 0.340 |
| Unmarried | 5 (8.5) | Ref |  | Ref |  |
